# Fungal infection mimicking COVID-19 infection – A case report

**DOI:** 10.1515/med-2022-0443

**Published:** 2022-04-28

**Authors:** Aleksandra Niemiec, Michał Kosowski, Marcin Hachuła, Marcin Basiak, Bogusław Okopień

**Affiliations:** Department of Internal Diseases, Allergology and Clinical Immunology, Medical University of Silesia, 40-752 Katowice, Poland; Department of Internal Medicine and Clinical Pharmacology, Medical University of Silesia, Medyków 18, 40-752 Katowice, Poland

**Keywords:** COVID-19 pneumonia, fungal pneumonia, differential diagnosis, high-resolution computed tomography

## Abstract

For the last 2 years, one of the most frequent causes of respiratory failure is coronavirus disease 2019 (COVID-19). The symptoms are not specific. Imaging diagnostics, especially high-resolution computed tomography, is a diagnostic method widely used in the diagnosis of this disease. It is important to emphasize that not only SARS-CoV-2 infection may manifest as interstitial pneumonia. Other diseases such as other viral, fungal, atypical bacterial pneumonia, autoimmune process, and even cancer can also manifest as ground-glass opacities or consolidations in the imaging of the lungs. In this case report, we described a patient who manifested many symptoms that seemed to be COVID-19. However, all performed antigen and polymerase chain reaction tests were negative. The diagnostics must have been extended. Microbiological and mycological blood cultures and sputum cultures were performed. Blood cultures were negative but in sputum, *Candida albicans* and *Candida glabrata* were identified. Targeted therapy with fluconazole was implemented with a satisfactory result. The patient was discharged from the hospital in a good general condition with no complaints.

## 1 Introduction

Acute respiratory distress syndrome is one of the most common reasons for internal ward admissions. Respiratory failure may be due to pulmonary or extra-pulmonary causes; it could be caused due to pneumonia, exacerbation of obstructive lung diseases, pulmonary edema, pleural effusion, and overdose of opioids or sedatives. It is necessary to take medical history at first, and perform an arterial blood gas (ABG) test and then chest imaging. These steps are crucial to make the preliminary diagnosis and to implement treatment [1].

For the last 2 years, one of the most frequent reasons for respiratory failure is coronavirus disease 2019 (COVID-19), a viral disease caused by a coronavirus (SARS-CoV-2). It is believed that the virus is acquired from a zoonotic source and is transmitted via airborne respiratory droplets [2]. The symptomatic phase, except for respiratory problems, manifests with fever, myalgia, and smell or taste disorders. The infection may be detected by a rapid antigen test; however, a reverse transcriptase-polymerase chain reaction (RT-PCR) test is needed to confirm the diagnosis [3,4]. The specific image of the lungs in high resolution computed tomography (HRCT) could be a useful diagnostic tool in the differential diagnosis. Many studies have also investigated the relationship between lung computerized tomography (CT) scans and patients’ prognosis [5,6]. Typical radiological manifestations presented in HRCT include ground-glass opacities (GGO), consolidations, crazy-paving, and reticular patterns [7].

However, in the time of numerous COVID-19 cases, it is very important that we cannot forget about the non-viral causes of pneumonia and the accompanying respiratory disorders. In our article, we would like to present the case of a patient treated in the internal ward due to pneumonia of initially unclear etiology.

## 2 Case report

A 73-year-old woman was urgently admitted to the Department of Internal Medicine and Clinical Pharmacology due to shortness of breath, fever, cough, and exercise intolerance. The patient denied direct contact with SARS-CoV-2-infected person in the last 14 days. However, she was not vaccinated against COVID-19. Medical interview showed symptoms of hypertension, diabetes type 2, hyperuricemia, chronic kidney failure in stage G3a according to Kidney Disease Improving Global Outcomes (KDIGO) and paroxysmal atrial fibrillation. Due to the coexistence of comorbidities, the patient had been ordered the following: ramipril, amlodipine, acetylsalicylic acid, chlorthalidone, glimepiride, spironolactone, metformin, allopurinol, and bisoprolol.

In the physical examination on admission, temperature of 36.1°C, blood pressure of 110/60 mmHg, respiratory rate of 24/min, and blood oxygen saturation of 81% were observed, and during lung auscultation, bilateral crackles, wheezing, and decreased respiratory sound in the base parts of lungs were examined. In the electrocardiography, atrial fibrillation with a ventricular rate of 100–130/min was observed. The examination revealed dyspnoea, tachypnoea, and increased work of additional respiratory muscles.

Laboratory tests showed white cells count 16.89 × 10^3^ cells/µL (reference range, 4–10 × 10^3^ cells/µL) with neutrophilia (92.4%) and lymphopenia (5.6%), hemoglobin count 8.5 g/dL (reference range, 11.5–15 g/dL), hematocrit 27.9% (reference range, 36–46%), platelets count 332 × 10^3^ cells/µL (130–400 × 10^3^ cells/µL), C-reactive protein concentration 34.6 mg/L (reference range: 0–5 mg/L), procalcitonin concentration 1.02 ng/mL (reference range: <0.5 ng/mL), interleukin-6 concentration 24.3 pg/mL (reference range: <7 pg/mL), serum creatinine concentration 1.06 mg/dL (reference range: 0.51–0.95 mg/dL), GFR MDRD: 54.01 mL/min (reference range: >60 mL/min), D-dimer concentration 1,040 ng/dL (reference range: <500 ng/dL). ABG showed pH 7.31 (reference range: 7.35–7.45), pCO2 44.3 mmHg (reference range: 35–46 mmHg), pO2 53.3 mmHg (reference range: 70–100 mmHg), SpO2 83% (reference range: >96%), lactate 3.15 mmol/L (reference range: <1.8 mmol/L), base excess (BE) 4.2 mmol/L (reference range: (−2) to 3 mmol/L), and {\text{HCO}}_{3}^{-}] 21.9 mmol/L (reference range, 21–26 mmol/L). Electrolytes, liver enzyme concentration, coagulation, and clinical urine tests were normal.

**Figure 1:**
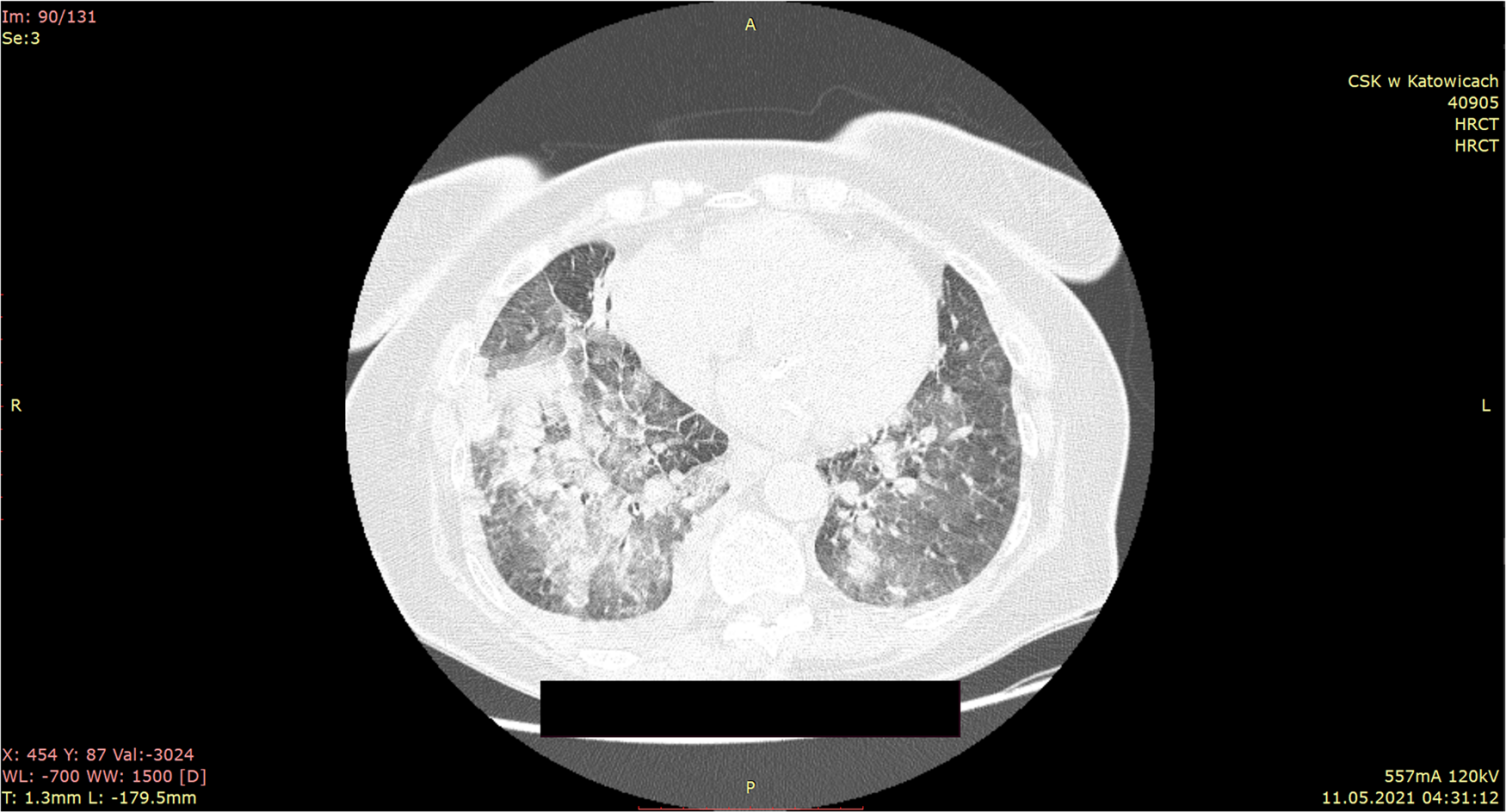
HRCT scans on hospital admission: bilateral, superimposed air space consolidations with GGO in lower and upper lobes, more marked on right are described.

Due to reported symptoms and laboratory results, COVID-19 was suspected. In order to assess the severity of pneumonia, it was decided to perform lung HRCT. In HRCT bilateral, superimposed air space consolidations with GGO in lowers and uppers lobes, more marked on right were described. Moreover, thickening of the interlobular septa was observed. There were mediastinal hilar lymphadenopathy and bilateral pleural effusion. The lesions accounted for 48% of the lungs. These HRCT findings were described as typical for COVID-19 pneumonia with a moderately advanced British Society of Thoracic Imaging (BSTI) score (Figure 1).

A nasopharyngeal swab sample for SARS-COV-2 was collected three times: two times for antigens tests and one for RT-PCR test. Both antigen tests and RT-PCR tests were negative. Microbiological and mycological blood cultures and sputum cultures were performed. Additionally, there were taken blood tests for the presence of IgM and IgG antibodies for *Mycoplasma pneumoniae*, *Chlamydophila pneumoniae*, SARS-CoV-2, Influenza A, B, and Respiratory Syncytial Virus, but they were all negative. Also, abdominal ultrasound examination and echocardiography were performed. No other outbreaks of potential infection were detected.

Before the results of the laboratory tests were obtained, due to the severe clinical condition, empirical therapy was introduced using ceftriaxone, levofloxacin, low molecular weight heparin, dexamethasone, budesonide, and ipratropium bromide.

Due to increasing respiratory failure despite oxygen therapy given and deteriorating results of ABG (pO2: 45.4 mmHg, SpO2: 79%) anesthesiologist’s consultation was performed. Prone position and switch type of oxygen therapy to high flow oxygen delivery 60 L/min FiO2: 0.9 was recommended. Moreover, it was recommended to take a nasopharyngeal swab in the SARS-CoV-2 infection one more time, and until the result is obtained, the patient should be treated like a COVID-19-positive person. Recommended procedures improved saturation of patient to 98%; however, this next swab was also negative.

Despite the suggestions of anesthesiologists, remdesivir and tocilizumab were not included in the treatment, suspecting other than viral etiology of pneumonia.

In the cultures following results were obtained: blood and urine were negative, whereas in sputum *Candida albicans* and *Candida glabrata* were identified. They were sensitive to fluconazole, amphotericin B, caspofungin, and micafungin. The antifungal drug Fluconazole 200 mg daily per os was added to therapy.

**Figure 2:**
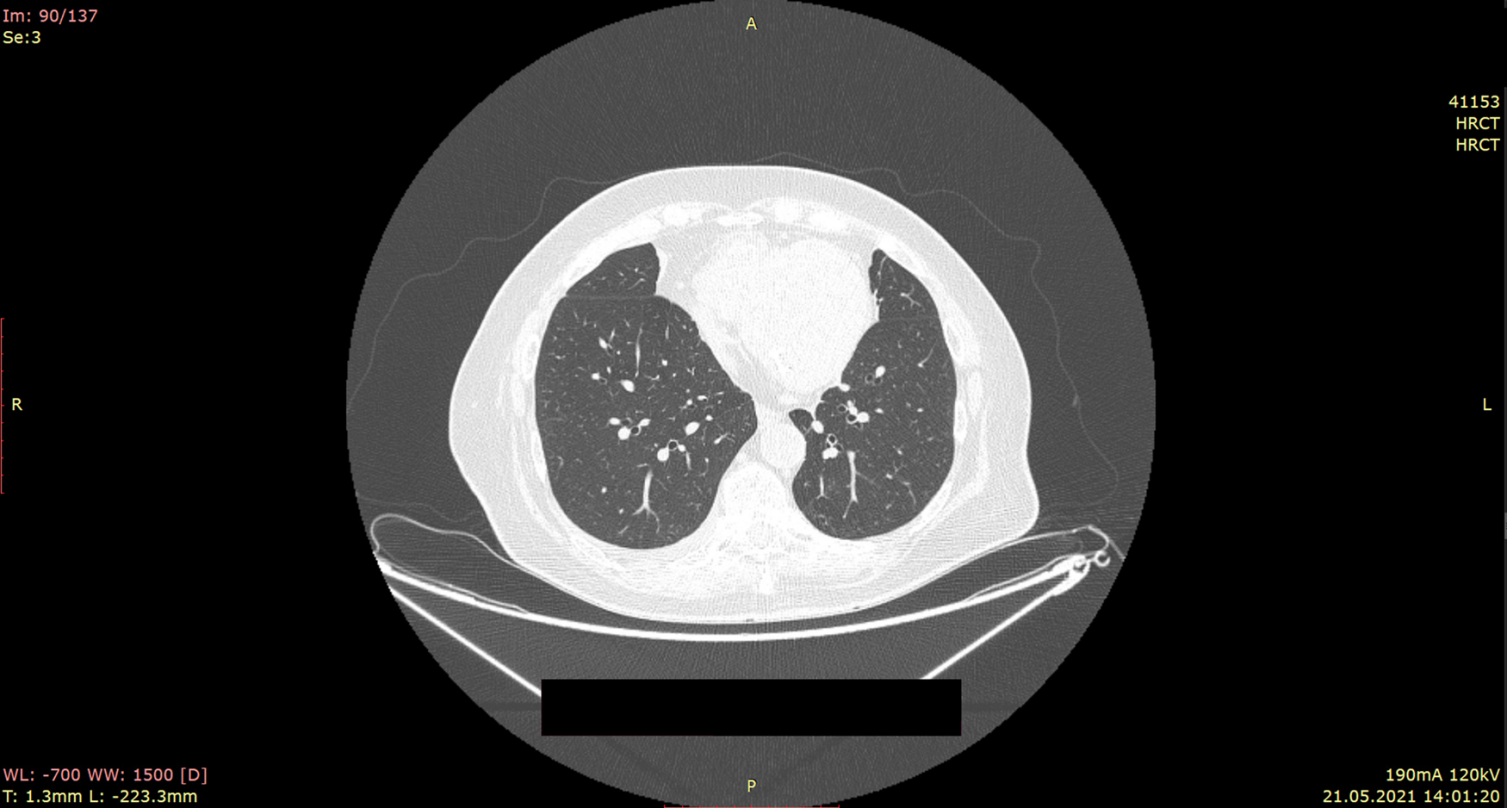
HRCT scans after antifungal treatment: regression of changes visible on admission is described.

During further hospitalization, improvement in the general state of the patient was observed. Oxygen therapy was gradually reduced. Atrial fibrillation was converted to regular sinus rhythm with ventricular action of 65 beats per minute. On the 10th day, the shortness of breath was not observed, and the patient did not require oxygen therapy (SpO2, 94%). Auscultation of the lung revealed a reduction of crackles and wheezing; the symmetrical respiratory sound was the dominant one. Laboratory determinants of inflammation were normalized. The control chest HRCT showed full withdrawal of inflammatory changes and regression of pleural effusion (Figure 2). At the base of the lungs, fibrous-atelectasis changes were reported.

The patient has been discharged from the hospital in a good general condition without any complaints and continued outpatient treatment with fluconazole.


**Institutional review board statement:** After consulting with the Bioethics Committee of the Medical University of Silesia in Katowice, ethical review and approval were waived because a case report does not require the approval of the bioethics committee.
**Ethical approval**: This is a description of a clinical case with a brief literature review. There was no formal research ethics approval required or no experimental intervention in routine care. Fully informed consent from the patient was obtained.

## 3 Discussion

During the COVID-19 pandemic, SARS-CoV-2 infection is mainly suspected as the reason for interstitial pneumonia. Due to the lack of specific symptoms, scientists from the beginning of the pandemic were looking for a test that would allow a clear diagnosis of the patient [8]. Over time, however, it turned out that the identification of infected people is not sufficient for their proper treatment. Tools that would be able to assess the severity of the disease, and thus the prognosis, have become necessary. These tools were the scales prepared by the researchers for the assessment of radiological examinations, both CT and chest X-ray [9].

Pan et al. divided lung involvement on chest CT into four stages. Stage 1 is dominated by GGO changes. In stage 2, additionally appear crazy paving pattern (CPP) and small consolidations. In the third phase, we observe the presence of consolidative foci sometimes with a halo sign. And in the fourth phase, called the absorption phase, GGO and linear consolidation are again described and interpreted as a sign of repair processes [10]. All the described phases and the observed changes in the CT image are summarized in Table 1.

**Table 1:** Radiological findings in COVID-19 pneumonia

Stage	Phase	Time (days)	Main radiological findings	Additional radiological findings (in every phase)
1	Early	0–4	GGO	Peripheral vessel widening
2	Progressive	5–8	GGO, CPP, and small consolidations	Halo sign
				Atoll sign or reversed halo sign
3	Peak	9–13	Consolidative foci	Overlapping of radiological findings in different phases
4	Absorption	≥14	GGO and linear consolidation	Rarity of: lymphadenopathies, pleuric effusions, pulmonary nodules

GGO, ground-glass opacities; CPP, crazy paving pattern.

However, other types of pneumonia may resemble that caused by SARS-CoV-2 in HRCT. That is the reason why other disease entities should be taken into consideration in the diagnostic process [11]. The symptoms and even imaging-study findings are not peculiar for viral infections [12]. Nevertheless, other viral, fungal, atypical bacterial pneumonia, autoimmune process, or cancer can also manifest as GGO in the imaging of the lungs [13,14]. There was even a case of a patient diagnosed with amiodarone-induced interstitial pneumonia described [15].

Acute respiratory distress syndromes with a cause other than COVID-19 have their own specific radiological features that are important in the differential diagnosis.

In the case of pneumonia caused by typical bacteria, a characteristic feature is the air bronchogram and the fact that the lesions do not exceed pleural cleavages [16]. Moreover, we can find in CT: centrilobular nodules, cavitations, pneumatoceles, mediastinal lymphadenomegalies, or pleural effusions [17,18]. Other types of lesions can be found in fungal pneumonia such as *Pneumocystis jiroveci* infection. In this type of pneumonia we see symmetrical, centroparenchymal and peripheral, confluent GGO, generally with subpleural sparing [19] and a predilection for the upper lobes [20], but we almost never observed CPP.

Cardiovascular disease is another group of diseases that can cause radiological features similar to COVID-19. For example, in pulmonary edema, we also can find in CT scans GGO, CPP, and consolidations but with different timing of occurrence with respect to COVID-19 pneumonia and also with accompanying cardiomegaly [21,22]. However, it should be remembered that due to the high similarity of CT images in this group of diseases to COVID-19, an anamnesis in the differential diagnosis plays a key role. Table 2 summarizes the most common disease entities requiring differentiation from COVID-19.

**Table 2:** Radiological features of pathologies in differential diagnosis with COVID-19 pneumonia

Pathologies		GGO	CPP	Consolidations	References
Infective pneumonia	Bacterial	R	A	C	[16,17,18]
	Viral	C	A	R	[17,23]
	Fungal	C	R	R	[19,20]
Cardiovascular	Acute pulmonary edema	C	C	C	[21,22]
	Acute pulmonary embolism	C	C	C	[24]
	Vasculities	C	C	C	[25]

GGO: ground-glass opacities, CPP: crazy paving pattern, C: common, R: rare, A: absent.

Because of this difficulty in the differential diagnosis, CT of the chest is not recommended for routine screening in patients under investigation for COVID-19 [26].

## 4 Conclusion

To sum up, it is necessary to remember that not only SARS-CoV-2 infection may manifest as severe pneumonia. Obviously, COVID-19 must also be taken into consideration, and special care should be provided during admission. Testing for SARS-CoV-2 must be performed in the first place to exclude the infection. However, other diseases or immunological disorders may cause similar symptoms. Proper diagnosis is crucial for subsequent proceedings and treatment. Particularly now, in times of the COVID-19 pandemic, physicians must not forget that differential diagnosis is an important part diagnostic process that allows us to make the right diagnosis and implement the right methods of therapy.
